# A Novel Endo-Polygalacturonase from *Penicillium oxalicum*: Gene Cloning, Heterologous Expression and Its Use in Acidic Fruit Juice Extraction

**DOI:** 10.4014/jmb.2112.12023

**Published:** 2022-01-07

**Authors:** Bo Lu, Liang Xian, Jing Zhu, Yunyi Wei, Chengwei Yang, Zhong Cheng

**Affiliations:** 1 Nanning University, 8 Longting Road, Nanning, Guangxi 530200, P.R. China; 2 National Engineering Research Center for Non-Food Biorefinery, Guangxi Key Laboratory of Biorefinery, Guangxi Academy of Sciences, 98 Daling Road, Nanning, Guangxi 530007, P.R. China

**Keywords:** Fruit juice, acidic fruit, endo-polygalacturonase, *Penicillium oxalicum*

## Abstract

An endo-polygalacturonase (endo-PGase) exhibiting excellent performance during acidic fruit juice production would be highly attractive to the fruit juice industry. However, candidate endo-PGases for this purpose have rarely been reported. In this study, we expressed a gene from *Penicillium oxalicum* in *Pichia pastoris*. The recombinant enzyme PoxaEnPG28C had an optimal enzyme activity at pH 4.5 and 45°C and was stable at pH 3.0–6.5 and < 45°C. The enzyme had a specific activity of 4,377.65 ± 55.37 U/mg towards polygalacturonic acid, and the *K*_m_ and *V*_max_ values of PoxaEnPG28C were calculated as 1.64 g/l and 6127.45 U/mg, respectively. PoxaEnPG28C increased the light transmittance of orange, lemon, strawberry and hawthorn juice by 13.9 ± 0.3%, 29.4 ± 3.8%, 95.7 ± 10.2% and 79.8 ± 1.7%, respectively; it reduced the viscosity of the same juices by 25.7 ± 1.6%, 52.0 ± 4.5%, 48.2 ± 0.7% and 80.5 ± 2.3%, respectively, and it increased the yield of the juices by 24.5 ± 0.7%, 12.7 ± 2.2%, 48.5 ± 4.2% and 104.5 ± 6.4%, respectively. Thus, PoxaEnPG28C could be considered an excellent candidate enzyme for acidic fruit juice production. Remarkably, fruit juice production using hawthorn as an material was reported for the first time.

## Introduction

Pectin is a biologically active macromolecule with a main chain constructed of polygalacturonic acid molecules connected by α-1,4 linkages [1]. Pectin mainly exists in the mesoglea of plant cells, and is important for the rigidity, flexibility and shape of the plant [2]. The group of enzymes that can degrade pectin is named pectinases [3], and pectinase reagent is widely used in fruit and vegetable juices production, animal feed, textiles, tea and coffee production, as well as in pulping and waste treatment [4]. In the fruit juice industry, the degradation of pectin results in higher yields, shorter production times, better impressions of the juice, lower production costs and higher profits. In the pectinase family, endo-polygalacturonase (endo-PGase), which randomly hydrolyzes the inner α-1,4 linkages of pectin, exhibits the highest rate of depolymerization of pectin [3, 5]. Thus, endo-PGase is highly important for the fruit juice industry [6].

The pH of most fruit juices is mildly acidic at 4.5–6.0, and such fruits are widely used in the production of fruit juices [7]. However, some fruits with lower pH (<4.5) are also rich in nutrients, have a unique smell and taste and even show probiotic function [8]. Most reported endo-PGases function well during mildly acidic juice production [9], but endo-PGases that function well in fruit juices with pH <4.5 have rarely been reported. Thus, the discovery of an endo-PGase that functions well during fruit juice production at low pH would be highly attractive to the fruit juice industry.

In previous reports, two endo-PGase encoding genes were found in the genome of *Penicillium oxalicum* 114-2 [10], and the recombinant endo-PGases (genes cloned from *P. oxalicum* CZ1028) produced by *Pichia pastoris* showed excellent performance in the production of fruit juice at pH >4.5 [9]. In this study, another gene from *P. oxalicum* CZ1028 encoding a putative endo-polygalacturonase was cloned and expressed in *P. pastoris*. The recombinant protein was purified and its enzyme characteristics were determined. Interestingly, this enzyme functioned well in fruit juice production at pH < 4.5.

## Materials and Methods

### Chemicals and Reagents

LA Taq DNA polymerase, DNA restriction endonuclease, T4 DNA ligase and RNA purification reagent RNAiso Plus were obtained from TaKaRa Bio Co. Ltd. (China). D-galacturonic acid, trigalacturonic acid, polygalacturonic acid (PGA, de-esterified), citrus pectin (<26% esterified) and apple pectin (50–75% esterified) were from Sigma-Aldrich Co. Ltd. (USA). Unless otherwise stated, all other chemicals were of analytical grade.

### Strains and Plasmid

The cultivation of *P. oxalicum* CZ1028 (CGMCC 3.15505) was conducted as described in a previous report [11]. Vector pPIC9K was used for gene cloning and expression in *P. pastoris* GS115, and *Escherichia coli* DH5α was used for construction of the recombinant plasmid pPIC9K-*PoxaEnPG28C*. Both *E. coli* and *P. pastoris* strains were cultivated using methods reported previously [12].

### Gene Cloning and Heterologous Expression

According to the genomic information of *P. oxalicum* 114-2, a putative protein EPS32977 was annotated as putative polygalacturonase [10]. Because the genomic information of different strains belonging to the same species is highly similar [5], a set of specific primers [5´-CGTACGTAGCACCCGCTCCCTCGCAGGT-3´ (SnaBI) and 5´-CCCCCTAGGTCAACACGAGGCCCCCGTAG-3´ (AvrII)] was designed according to the gene sequence encoding the putative protein EPS32977 to amplify the cDNA fragment without the signal peptide-encoding region of the corresponding gene. The amplification conditions were 94°C for 5 min; followed by 30 cycles of 94°C for 30 s, 55°C for 30 s and 72°C for 1 min; and a final step of 72°C for 10 min. The amplified cDNA was inserted into the vector pPIK9K, and plasmid transformation and gene expression were conducted according to a previous report [12].

### Protein Purification and Determination

The recombinant protein PoxaEnPG28C in the culture broth was concentrated and applied to size exclusion chromatography using a HiLoad 16/600 Superdex 75 column (GE Co., Ltd., Sweden). Sodium dodecyl sulphate polyacrylamide gel electrophoresis [SDS-PAGE, 5% (w/v) stacking gel and 10% (w/v) separating gel] was used to determine the purity and molecular weight of the enzyme [13], and the Bradford method with bovine serum albumin protein as the standard was used to determine the protein concentration [14]. The purified enzyme was desalted by dialyzing against ultrapure water, then biochemically characterized and used for fruit juice extraction.

### Enzyme Assay

The enzyme activity of PoxaEnPG28C was determined by the same method as used for the previously reported enzymes PoxaEnPG28A [12] and PoxaEnPG28B [12], and the concentration of the released reducing sugar was calculated using a standard curve constructed with D-galacturonic acid as the standard. One unit of enzyme activity was defined as the liberation of 1 μmol of reducing sugar from the enzyme-catalyzed reaction system in 1min.

### Mode of Action of PoxaEnPG28C

The purified and desalted PoxaEnPG28C was incubated with 0.5% PGA at 45°C for 0.25, 0.5, 1, 2, 8, 12, 18, and 24 h, and then the liberated products were analyzed by thin-layer chromatography according to the previously reported method [11].

### Effects of pH and Temperature on the Enzyme Activity and Stability

To determine the optimal pH, the enzyme activity of PoxaEnPG28C was determined at different pH values and at 50°C; for the optimal temperature, the enzyme activity of PoxaEnPG28C was determined at different temperatures (at the optimal pH). The highest enzyme activity was defined as 100%, and other enzyme activities were calculated as relative values.

For pH stability, 0.1 mL of PoxaEnPG28C was mixed with 0.9 mL of buffers with different pH values (0.1 M citric acid-Na_2_HPO_4_ for pH 3.0–7.0 and 0.1 M Tris-HCl for pH 7.0–9.0) at 4°C for 24 h, and then the residual activities were determined. For the thermal stability, PoxaEnPG28C was incubated at 45, 50, 55, and 60°C for 15, 30, 45, and 60 min, and the residual enzyme activities were measured. The enzyme activity of un-incubated enzyme was defined as 100%, and the residual enzyme activities were calculated as relative values.

### Substrate Specificity and Michaelis-Menten Constant of the Enzyme

For substrate specificity, the enzyme activities of PoxaEnPG28C against 0.5% (w/v) PGA, citrus pectin and apple pectin were determined under optimal reaction conditions (pH 4.5 and 45°C). The enzyme activity against PGA was defined as 100%, and other enzyme activities against other substrates were calculated as relative values. For the Michaelis-Menten constant, enzyme activities of PoxaEnPG28C against 0.2–2.0 g/l PGA were determined under the optimal reaction conditions (pH 4.5 and 45°C). The *K*_m_ and *V*_max_ values were then obtained by calculation using the Lineweaver-Burk plotting method.

### Effects of Metal Ions on Enzyme Activity

The enzyme activities of PoxaEnPG28C were determined in the presence of each of the following metal ions: 2mM Na^+^, K^+^, Mg^2+^, Ca^2+^, Mn^2+^, Ba^2+^, Co^2+^, Zn^2+^, Fe^2+^, or Cu^2+^. The enzyme activity without extra additive was defined as 100%, and other enzyme activities were calculated as relative values.

### Use of PoxaEnPG28C for Acidic Fruit Juice Extraction

Fresh oranges, lemons, strawberries and hawthorn were purchased from the local market. Oranges and lemons were peeled, and the hawthorns were cored. Fresh fruits were then mixed with ultrapure water and pulped. Twenty-five grams of pulp were treated with 0 or 100 U of PoxaEnPG28C per gram of pulp (at pH 4.5 and 45°C) for 2.5 h, and then centrifuged in a tubular filter (400 mesh) at 500 rpm for 2 min. The filtered juice was centrifuged at 5,000 ×*g* for 10 min, and the parameters of the supernatant were determined according to previously reported methods [12]. The parameters of the juices without addition of PoxaEnPG28C were set as the control values (100%).

### Sequence Analysis and Nucleotide Sequence Accession Number

The SignalP 4.1 online server (http://www.cbs.dtu.dk/services/SignalP/) and SMART online server (http://smart.embl-heidelberg.de/) were used to predict the signal peptide and catalytic domain, respectively. ExPASy online server (http://web.expasy.org/protparam/) was used to calculate the hypothetical molecular weight of the protein. DNAMAN 6.0 software was used to carry out the multiple sequence alignment. The nucleotide sequence of the gene encoding PoxaEnPG28C has been deposited in the GenBank database under the Accession No. MZ614864.

## Results and Discussion

### Gene Cloning, Heterologous Expression and Identification of the Recombinant Protein

**Fig. 1:**
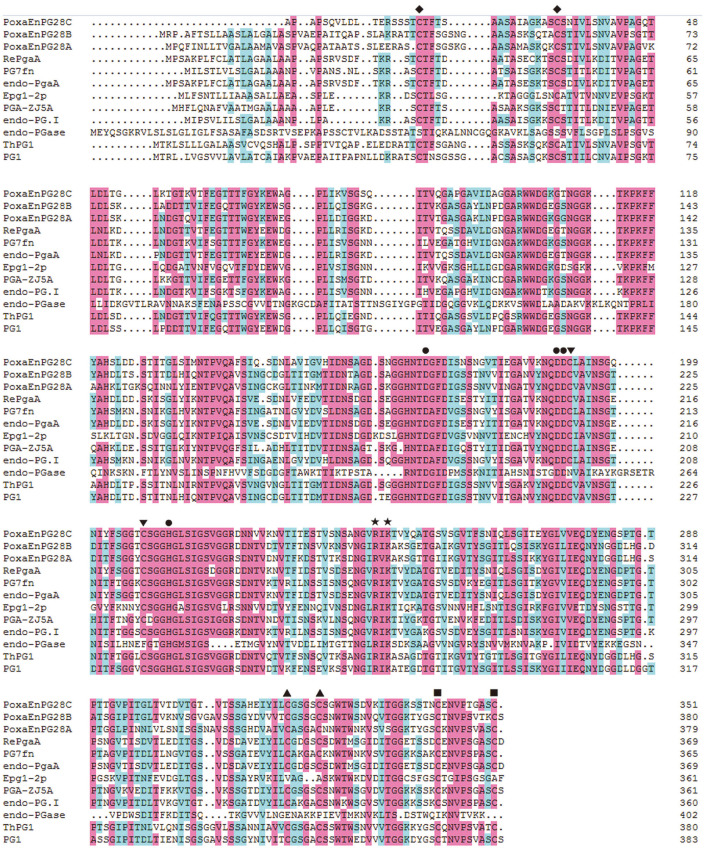
Multiple homology alignment of PoxaEnPG28C with other GH 28 endo-PGases. The amino acid residue sequences of PoxaEnPG28C and 11 functionally characterized endo-PGases of PoxaEnPG28B (from *P. oxalicum* CZ1028, GenBank Accession No. APZ75903), PoxaEnPG28A (from *P. oxalicum* CZ1028, GenBank Accession No. ANS70886), RePgaA (from *A. niger* JL-15, GenBank Accession No. AGV40780), PG7fn (from *T. arenaria*, GenBank Accession No. AIZ95162), endo-PgaA (from *A. niger* SC323, GenBank Accession No. AJD09825), Epg1-2p (from *Kluyveromyces marxianus* CECT1043, GenBank Accession No. AAR84199), PGA-ZJ5A (from *A. niger* ZJ5, GenBank Accession No. AQT01640), endo-PG I (from *Achaetomium* sp. Xz8, GenBank Accession No. AGR51994), endo-PGase (from *Pectobacterium carotovorum*, GenBank Accession No. WP_039543807), ThPG1 (from *Trichoderma harzianum* T34, GenBank Accession No. CAM07141) and PG1 (from *Penicillium occitanis*, GenBank Accession No. ABS50231) were used for multiple homology alignment. Amino acid residues showing identities of higher than 80% and 40% are shaded in light red and light green, respectively. The Asp^169^, Asp^190^, Asp^191^ and His^212^ of PoxaEnPG28C, which were expected to be the catalytic residues, are marked by solid circles. The Arg^245^ and Lys^247^ of PoxaEnPG28C, which were expected to be involved into the binding of substrate, are marked by solid stars. The Cys^18^ and Cys^33^, Cys^192^ and Cys^208^, Cys^318^ and Cys^323^, and Cys^342^ and Cys^351^ of PoxaEnPG28C, which were expected to form disulfide bonds, are marked by solid regular triangles, solid downward triangles, solid upward triangles and solid squares, respectively.

**Fig. 2:**
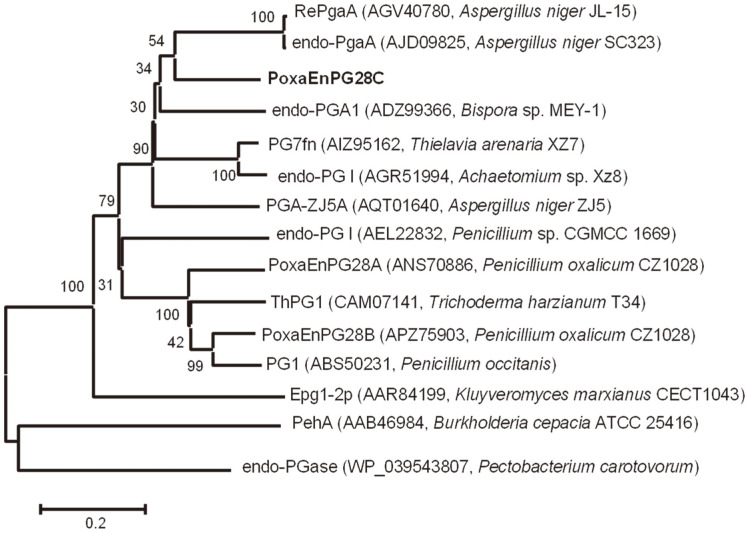
Phylogenetic tree of amino acids of PoxaEnPG28C with reported endo-PGases. The phylogenetic tree of the amino acids of PoxaEnPG28C with other reported endo-PGases (from fungi, yeast and bacteria) was constructed by using MEGA software. PoxaEnPG28C is boldfaced.

After prediction using the SMART online tool, a putative signal peptide including the initial 20 amino acids and a GH 28 catalytic domain were found in the putative protein EPS32977 of *P. oxalicum* 114-2 [10]. The amino acid sequence encoded by the amplified cDNA from *P. oxalicum* CZ1028 showed 100% identity with the putative protein EPS32977 of *P. oxalicum* 114-2, and showed 78.35% identity with a functional identified endo-PGase from *Penicillium janthinellum* IF0 7719 [15]. In comparison with a functionally mutated endo-PGase [16], Asp^169^, Asp^190^, Asp^191^, and His^212^ were expected to be the catalytic residues of the amplified putative endo-PGase, and Arg^245^ and Lys^247^ of the putative endo-PGase were expected to be involved in the binding of substrate. Four putative disulfide bonds were formed by Cys^18^ and Cys^33^, Cys^192^ and Cys^208^, Cys^318^ and Cys^323^, and Cys^342^ and Cys^351^ of the amplified putative endo-PGase (Fig. 1). Compared with two previously reported endo-PGases from *P. oxalicum* CZ1028 (PoxaEnPG28A [12] and PoxaEnPG28B-Pp [9]), PoxaEnPG28C had a closer phylogenetic relationship with endo-PGases from *Aspergillus niger* (RePgaA from *A. niger* JL-15 [17] and endo-PgaA from *A. niger* SC323 [18]), emphasizing the diversity of the genome of *P. oxalicum* (Fig. 2).

**Fig. 3:**
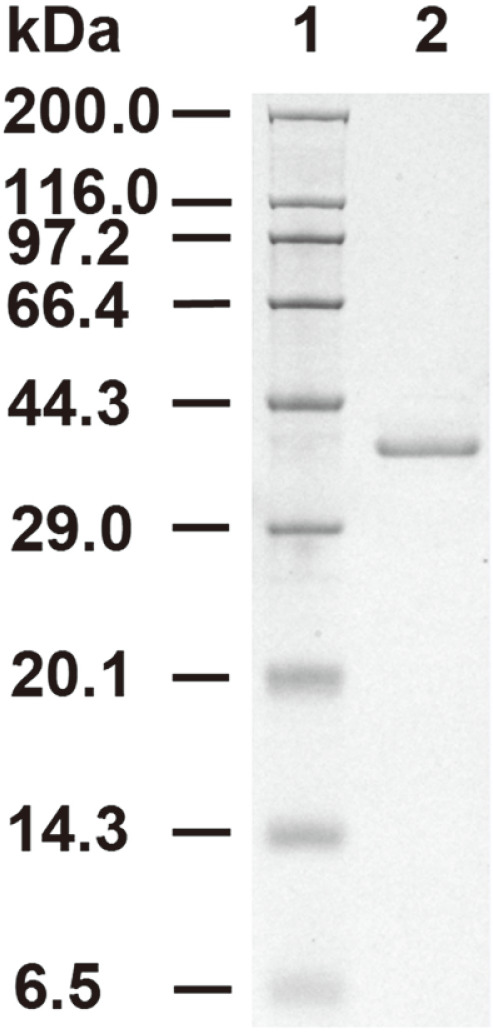
SDS-PAGE analysis of PoxaEnPG28C. Lane 1, protein molecular weight ladder; lane 2, purified recombinant protein PoxaEnPG28C.

After 4 days of cultivation, the enzymatic activity of the pectinase in the culture broth of the recombinant *P. pastoris* reached 1057.98 ± 28.31 U/ml, which was higher than the activities of endo-PGI produced by *P. pastoris* (6.2 U/ml) [19], endo-PGA1 produced by *P. pastoris* (50 U/ml) [20], PG1 produced by Saccharomyces cerevisiae (50 U/ml) [21], and PG7fn produced by *P. pastoris* (678.1 U/ml) [22]. After purification, a protein showing a sharp band at about 36 kDa on the SDS-PAGE gel was obtained (Fig. 3), and the apparent molecular weight matched the theoretical molecular weight of 35.87 kDa.

**Fig. 4:**
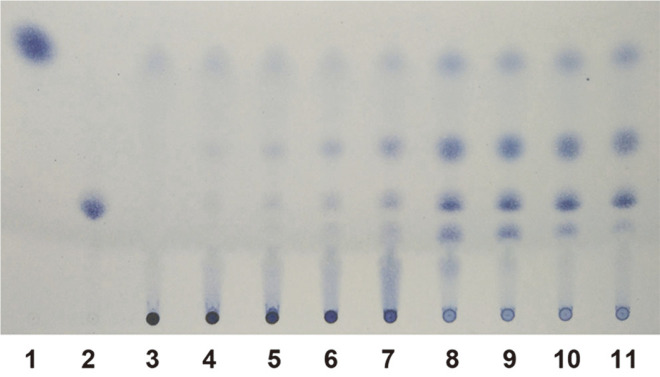
Thin-layer chromatography analysis of liberated products from the hydrolysis of polygalacturonic acid by PoxaEnPG28C. Lane 1, galacturonic acid; lane 2, trigalacturonic acid; lanes 3 to 11, liberated products of PGA by PoxaEnPG28C for 0, 0.25, 0.5, 1, 2, 8, 12, 18, and 24 h.

At the early stage of hydrolysis, galacturonic acid, digalacturonic acid and trigalacturonic acid appeared among the products (Fig. 4). Tetramers and pentamers of galacturonic acid appeared later, but as the hydrolysis time became prolonged, the amounts of tetramers and pentamers of galacturonic acid decreased. Meanwhile, the amount of galacturonic acid did not increase during the hydrolysis process, indicating the bond connecting the terminal galacturonic acid was not the prime target of the recombinant enzyme. The variations in the amounts of mono- and oligo-galacturonic acid in the hydrolysis product confirmed an endo-acting pattern of the recombinant enzyme [3]. Because the enzyme was the third reported endo-PGase from *P. oxalicum* [12], it was named as PoxaEnPG28C.

### Effects of pH and Temperature on the Enzyme Activity and Stability

**Fig. 5:**
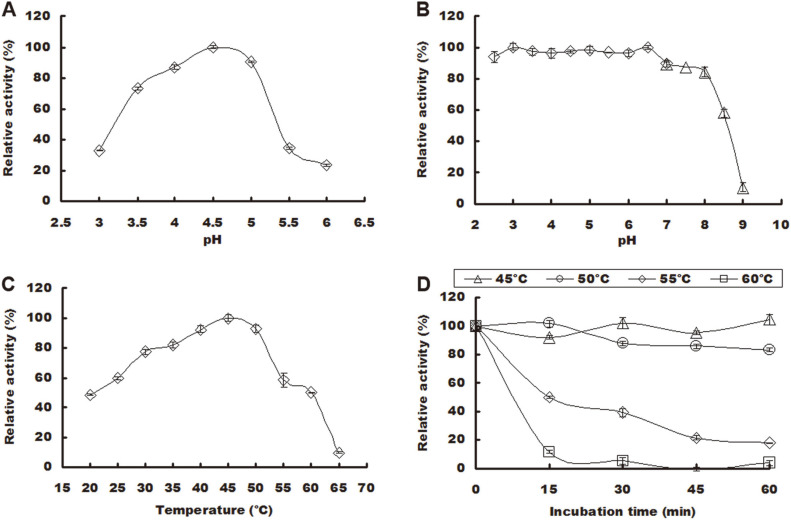
Effects of pH and temperature on the enzyme activity and stability of the purified PoxaEnPG28C. (**A**) Effect of pH on enzyme activity. (**B**) Effect of pH on enzyme stability. (**C**) Effect of temperature on enzyme activity. (**D**) Effect of temperature on enzyme stability. Temperatures were 45°C (open triangle), 50°C (open circle), 55°C (open diamond) and 60°C (open square). Error bars represent the standard deviation of three repeats.

PoxaEnPG28C exhibited its highest enzyme activity at pH 4.5 and greater than 90% enzyme activity at pH 4.0 and 5.0 (Fig. 5A); it was also stable within the acidic pH range of 3.0–6.5 (Fig. 5B). The range of conditions under which PoxaEnPG28C exhibited high relative activity (pH 4.0 to 5.0) covered some acidic fruit juices such as lemon (pH 3.5) [23], apple (pH 3.8) [19], grape (pH 3.4 to 3.9) [24], mango (pH 4.52) and pitaya (pH 4.56) [12]. PoxaEnPG28C was a mesophilic endo-PGase that exhibited its highest enzyme activity at 45°C and greater than 90% enzyme activity at 40–50°C (Fig. 5C), and its optimal temperature was similar to that of PgaB (40°C) [25], endo-PGI (45°C) [26] and endo-PGA1 (50°C) [20]. PoxaEnPG28C was quite stable at its optimal temperature (45°C), and lost less than 20% of its enzyme activity after incubation for 1 h at 50°C (Fig. 5D).

Endo-PGases (previously reported as PoxaEnPG28A [12] and PoxaEnPG28B-Pp [9] and herein PoxaEnPG28C) from *P. oxalicum* CZ1028 have different enzymatic characteristics. Similarly, several endo-PGases have been reported in *A. niger* with different enzymatic characteristics [17, 18, 27-32]. Compared with PoxaEnPG28A (optimal pH of 5.5) [12] and PoxaEnPG28B-Pp (optimal pH of 5.0) [9], PoxaEnPG28C had a lower optimal pH (pH 4.5) and might be more suitable in the production of acidic fruit juices.

### Substrate Specificity and Michaelis-Menten Constant of the Enzyme

PoxaEnPG28C showed higher enzyme activity towards PGA (4,377.65 ± 55.37 U/mg, 100.00 ± 1.26%) than towards citrus pectin (1,321.32 ± 43.13 U/mg, 30.18 ± 0.99%) and apple pectin (704.39 ± 51.61 U/mg, 16.09 ± 1.18%), indicating that its hydrolysis activity was hindered by the methylation of the backbone [12]. As the concentration of PGA increased, the specific enzyme activity of PoxaEnPG28C increased and then remained constant (Fig. S1A). Moreover, the *K*_m_ and *V*_max_ values were calculated to be 1.64 g/l and 6127.45 U/mg, respectively (Fig. S1B). The *K*_m_ of PoxaEnPG28C was in the range of previously reported *K*_m_ values of endo-PGases (from 0.32 g/l to 19.5 g/l) [12]. The *V*_max_ value of PoxaEnPG28C was higher than those of some reported endo-PGases, 55.55 U/mg [33], 103.58 U/mg [25] and 240 U/mg [34], but lower than that of PoxaEnPG28B-Pp (77,882.2 U/mg) [12].

### Effects of Metal Ions on Enzyme Activity

Ba^2+^ reduced the enzyme activity of PoxaEnPG28C to 80.88 ± 1.76 (Table S1), which was higher than that of PG2 (0%) [23] and lower than that of PoxaEnPG28A (93.7 ± 0.7%) [12]. Similar to that of PoxaEnPG28A [12], the enzyme activity of PoxaEnPG28C was also strongly inhibited by Mn^2+^. Different from PG2 (2.48%) [23], endo-PGA1 (22.2±2.9%) [20] and PoxaEnPG28A (19.7 ± 4.6%) [12], PoxaEnPG28C exhibited 88.78 ± 1.59% enzyme activity in the presence of 2 mM Cu^2+^. The enzyme activity of PoxaEnPG28C was partially inhibited by Ca^2+^, K^+^, Na^+^, Ni^2+^, and Zn^2+^. The slight inhibitory effects of Co^2+^ and Mg^2+^ were similar to those of PoxaEnPG28A [12]; however, the inhibitory effect of Co^2+^ was obvious in some other cases such as PoxaEnPG28-Pp (67.7 ± 0.4%) [12] and PG2 (0.31%) [23].

### Use of PoxaEnPG28C for Acidic Fruit Juice Extraction

The pH of oranges (*Citrus reticulata* Blanco) used in this study (pH 4.44) was different from that of a previous report of pH 3.5 [23]. PoxaEnPG28C reduced the viscosity by 25.7 ± 1.6%, increased the light transmittance by 13.9 ± 0.3% and increased the yield by 24.5 ± 0.7% (Table 1). In a previous report, a purified endo-PGase PG1 also increased the yield of citrus juice from 2.5 ml to 3.75 ml [35]. Lemon [*Citrus limon* (L.) Burm. f.] belongs to the same genus as orange, but it is more acidic than orange. The pH of lemons used in this study (pH 2.58) was lower than that of a previous report of 3.50 [23]. PoxaEnPG28C reduced the viscosity by 52.0 ± 4.5%, increased the light transmittance by 29.4 ± 3.8% and increased the yield by 12.7 ± 2.2% (Table 1). In previous reports, endo-PGases PG2 and PgaB increased the light transmittance of lemon juice by 43% [23] and about by 40% [25], respectively. However, some other important improvements in the juice such as a reduction in viscosity and an incremental yield of the juice were not mentioned in those reports [9].

The pH of strawberries (*Fragaria vesca*) used in this study (pH 3.41) was close to that of a report of 3.9 [36]. PoxaEnPG28C reduced the viscosity by 48.2 ± 0.7%, increased the light transmittance by 95.7 ± 10.2% and increased the yield by 48.5 ± 4.2% (Table 1). The performance of PoxaEnPG28C was better than those of other endo-PGases including NfPGI (reduction of viscosity: 32.4%; increment of light transmittance: 28.72%; increment of yield: 6%) and NfPGII (reduction of viscosity: 20.6%; increment of light transmittance: 46.41%; increment of yield: 9%) [36]. Moreover, the enzyme dosage quantity of PoxaEnPG28C was 100 U/g juice pulp, which was lower than the dosages of 7,500 U/ml (for NfPG I) and 3,000 U/ml (for NfPGII) /g juice pulp (the density of strawberry pulp was 1.02 g/ml) [36].

Hawthorn fruit (*Crataegus pinnatifida* Bunge) is high in nutritional value; it contains (100 g fresh weight basis) 23.688 g citric acid, 18.378 g fructose, 13.893 g glucose and 9.418 mg vitamin C, and has a unique taste [37]. However, it is seldom used to produce juice. PoxaEnPG28C reduced the viscosity of hawthorn juice by 80.5 ± 2.3%, increased the light transmittance by 79.8 ± 1.7% and increased the yield by 104.5 ± 6.4% (Table 1). The excellent performance of PoxaEnPG28C might aid the development of hawthorn juice as a commercial product.

Endo-Pgase should function well in several types of fruit juice since the varied hydrolysis environment of pectin is a challenge for the enzyme [7]. The pH values of fruit pulp used in this study were different from previous reports, which might be due to the differences in the cultivars and the maturity of the fruits [7]. However, the differences in catalytic environment in the different fruit juices go well beyond pH and include different cell-wall compositions and polymer structures, which can have a major influence on the catalytic efficiency of endo-Pgase. The degradation of natural pectin in fruits is much more difficult than the degradation of purified pectin because natural pectin takes part in and is embedded in the network of the plant cell wall [2]. The structure of plant cell wall and catalytic environments of juices differ from those of fruits [38] and are dependent on the ripening periods of the fruit [39]. Thus, compared with other endo-Pgases which only function well in one kind of fruit juice, the performance of PoxaEnPG28C in four acidic fruit juice preparations emphasizes the considerable application potential of this enzyme (Table 2).

The efficient production of endo-Pgases is highly attractive in the industry; however, *P. pastoris* is not a suitable host because fine processes (*e.g.*, O_2_, inducement, and high cell density) [26] and high-cost materials (*e.g.*, yeast extract, peptone, and glycerol) are required for the production of recombinant endo-Pgases by *P. pastoris* [9, 12, 17, 19, 20, 22, 26, 27, 40]. In contrast, filamentous fungal strains (*e.g.*, *A. niger*) might be more suitable for industrial enzyme production [41]. *A. niger* strains produce cellulase, xylanase, amylase, and proteinase [42-44], which allow the utilization of inexpensive agricultural residues (*e.g.*, wheat bran, corn core, and peanut cake) for enzyme production [45, 46]. In future studies, a recombinant *A. niger* strain will be used for the low-cost production of PoxaEnPG28C [47].

In this study, a gene encoding a putative endo-polygalacturonase was cloned from *P. oxalicum* and expressed in *P. pastoris*, and the recombinant protein PoxaEnPG28C was purified and identified as an endo-polygalacturonase. The enzymatic characteristics of PoxaEnPG28C were determined, and it was found to be an acidic enzyme. Remarkably, PoxaEnPG28C functioned well in orange, lemon, strawberry and hawthorn juice production. Thus, PoxaEnPG28C could be considered an excellent candidate enzyme for acidic fruit juice production.

## Supplemental Materials

Supplementary data for this paper are available on-line only at http://jmb.or.kr.

## Figures and Tables

**Table 1:** Application of PoxaEnPG28C in fruit juice production.

Fruits	Initial pH of juice	Reduction of viscosity (%)	Increment of light transmittance (%)	Increment of yield (%)
Orange	4.44	25.7 ± 1.6	13.9 ± 0.3	24.5 ± 0.7
Lemon	2.58	52.0 ± 4.5	29.4 ± 3.8	12.7 ± 2.2
Strawberry	3.41	48.2 ± 0.7	95.7 ± 10.2	48.5 ± 4.2
Hawthorn	2.92	80.5 ± 2.3	79.8 ± 1.7	104.5 ± 6.4

**Table 2:** Reported application of endo-Pgases in acidic fruit juice production.

Enzyme name	Source strain	Optimal pH of enzyme	Fruit	Initial pH of juice	Performance of enzyme	Reference
PoxaEnPG28C	*Penicillium oxalicum* CZ1028	4.5	Orange	4.44	RV^a^: 25.7±1.6 %, ILT^b^: 13.9±0.3 %, IY^c^: 24.5±0.7 %.	This study
			Lemon	2.58	RV^a^: 52.0±4.5 %, ILT^b^: 29.4±3.8 %, IY^c^: 12.7±2.2 %.	
			Strawberry	3.41	RV^a^: 48.2±0.7 %, ILT^b^: 95.7±10.2 %, IY^c^: 48.5±4.2 %.	
			Hawthorn	2.92	RV^a^: 80.5±2.3 %, ILT^b^: 79.8±1.7 %, IY^c^: 104.5±6.4 %.	
NM^d^	*Penicillium occitanis* CT1	6.0	Lemon	3.50	ILT^b^: 43.00 %, increased the reducing sugars by 17%, reduced color by 34%.	[23]
endo-PG I	*Penicillium* sp. CGMCC 1669	3.5	Apple	3.8	RV^a^: 4.5 %, ILT^b^: 71.8 %.	[19]
endo-PGA1	*Bispora* sp. MEY-1	3.5	Apple	4.0	RV^a^: 7.56 %, ILT^b^: 84.46 %.	[20]
TePG28b	*Talaromyces leycettanus* JCM 12802	3.5	Grape	3.4-3.9	ILT^b^: 69 %	[24]

RV^a^: Reduction of viscosity (%); ILT: Increment of light transmittance (%); IY: Increment of yield (%).NM^d^: Not mentioned in the reference.

## References

[1] Willats WGT, Knox JP, Mikkelsen JD (2006). Pectin: new insights into an old polymer are starting to gel. Trends Food Sci. Technol..

[2] Voragen AGJ, Coenen G-J, Verhoef RP, Schols HA (2009). Pectin, a versatile polysaccharide present in plant cell walls. Struct. Chem..

[3] Jayani RS, Saxena S, Gupta R (2005). Microbial pectinolytic enzymes: a review. Process Biochem..

[4] John J, Kaimal KKS, Smith ML, Rahman PKSM, Chellam PV (2020). Advances in upstream and downstream strategies of pectinase bioprocessing: a review. Int. J. Biol. Macromol..

[5] Xian L, Wang F, Yin X, Feng JX (2016). Identification and characterization of an acidic and acid-stable endoxyloglucanase from *Penicillium oxalicum*. Int. J. Biol. Macromol..

[6] Kashyap DR, Vohra PK, Chopra S, Tewari R (2001). Applications of pectinases in the commercial sector: a review. Bioresour. Technol..

[7] Lozano JE (2006). Fruit manufacturing: scientific basis, engineering properties, and deteriorative reactions of technological importance (Food Engineering Series).

[8] Saúco VG, Herrero M, Hormaza JI (2014). Tropical and Subtropical Fruits. Horticulture: Plants for people and places, Volume 1.

[9] Cheng Z, Xian L, Chen D, Lu J, Wei YT, Du LQ (2020). Development of an innovative process for high-temperature fruit juice extraction using a novel thermophilic endo-polygalacturonase from *Penicillium oxalicum*. Front. Microbiol..

[10] Liu GD, Zhang L, Wei XM, Zou G, Qin YQ, Ma L (2013). Genomic and secretomic analyses reveal unique features of the lignocellulolytic enzyme system of *Penicillium decumbens*. PLoS One.

[11] Cheng Z, Chen D, Lu B, Wei YT, Xian L, Li Y (2016). A novel acid-stable endo-polygalacturonase from *Penicillium oxalicum* CZ1028: purification, characterization and application in the beverage industry. J. Microbiol. Biotechnol..

[12] Cheng Z, Chen D, Wang QY, Xian L, Lu B, Wei YT (2017). Identification of an acidic endo-polygalacturonase from *Penicillium oxalicum* CZ1028 and its broad use in major tropical and subtropical fruit juices production. J. Biosci. Bioeng..

[13] Laemmli UK (1970). Cleavage of structural proteins during the assembly of the head of bacteriophage T4. Nature.

[14] Bradford MM (1976). A rapid and sensitive method for the quantitation of microgram quantities of protein utilizing the principle of protein-dye binding. Anal. Biochem..

[15] Ishida Y, Kakibuchi K, Hirao Y, Izumori K (1997). Cloning and characterization of a polygalacturonase-encoding gene from *Penicillium janthinellum*. J. Ferment. Bioeng..

[16] van Santen Y, Benen JAE, Schröter K-H, Kalk KH, Armand S, Visser J (1999). 1.68-A crystal structure of endopolygalacturonase II from *Aspergillus niger* and identification of active site residues by site-directed mutagenesis. J. Biol. Chem..

[17] Liu MQ, Dai XJ, Bai LF, Xu X (2014). Cloning, expression of *Aspergillus niger* JL-15 endo-polygalacturonase A gene in *Pichia pastoris* and oligo-galacturonates production. Protein Expr. Purif..

[18] Zhou HX, Li X, Guo MY, Xu QR, Cao Y, Qiao DR (2015). Secretory expression and characterization of an acidic endopolygalacturonase gene from *Aspergillus niger* SC323 in *Saccharomyces cerevisiae*. J. Microbiol. Biotechnol..

[19] Yuan P, Meng K, Huang HQ, Shi PJ, Luo HY, Yang PL (2011). A novel acidic and low-temperature-active endo-polygalacturonase from *Penicillium* sp. CGMCC 1669 with potential for application in apple juice clarification. Food Chem..

[20] Yang J, Luo HY, Li J, Wang K, Cheng HP, Bai YG (2011). Cloning, expression and characterization of an acidic endopolygalacturonase from *Bispora* sp. MEY-1 and its potential application in juice clarification. Process Biochem..

[21] Rojas NL, Ortiz GE, Baruque DJ, Cavalitto SF, Ghiringhelli PD (2011). Production of heterologous polygalacturonase I from *Aspergillus kawachii* in *Saccharomyces cerevisiae* in batch and fed-batch cultures. J. Ind. Microbiol. Biotechnol..

[22] Tu T, Meng K, Huang HQ, Luo HY, Bai YG, Ma R (2014). Molecular characterization of a thermophilic endo-polygalacturonase from *Thielavia arenaria* XZ7 with high catalytic efficiency and application potential in the food and feed industries. J. Agr. Food Chem..

[23] Sassi AH, Tounsi H, Trigui-Lahiani H, Bouzouita R, Romdhane ZB, Gargouri A (2016). A low-temperature polygalacturonase from *P. occitanis*: characterization and application in juice clarification. Int. J. Biol. Macromol..

[24] Li YQ, Wang Y, Tu T, Zhang DD, Ma R, You S (2017). Two acidic, thermophilic GH28 polygalacturonases from *Talaromyces leycettanus* JCM 12802 with application potentials for grape juice clarification. Food Chem..

[25] Tan HD, Yang GJ, Chen W, Liu QS, Li KK, Yin H (2020). Identification and characterization of thermostable endopolygalacturonase II B from *Aspergillus luchuensis*. J. Food Biochem..

[26] Tu T, Meng K, Bai YG, Shi PJ, Luo HY, Wang YR (2013). High-yield production of a low-temperature-active polygalacturonase for papaya juice clarification. Food Chem..

[27] Wang JJ, Zhang YH, Qin X, Gao LY, Han B, Zhang DQ (2017). Efficient expression of an acidic endo-polygalacturonase from *Aspergillus niger* and its application in juice production. J. Agr. Food Chem..

[28] Kant S, Vohra A, Gupta R (2013). Purification and physicochemical properties of polygalacturonase from *Aspergillus niger* MTCC 3323. Protein Exp. Purif..

[29] Juwon AD, A AF, Kayode OA (2012). Purification, characterization and application of polygalacturonase from *Aspergillus niger* CSTRF. Malaysian J. Microbiol..

[30] Dinu D, Nechifor MT, Stoian G, Costache M, Dinischiotu A (2007). Enzymes with new biochemical properties in the pectinolytic complex produced by *Aspergillus niger* MIUG 16. J. Biotechnol..

[31] Guo CT, Xue WM, Chen TB, Deng WH, Rao PF (2002). Purification and partial characterization of an endo-polygalacturonase from *Aspergillus niger*. J. Food Biochem..

[32] Singh SA, Rao AGA (2002). A simple fractionation protocol for, and a comprehensive study of the molecular properties of, two major endopolygalacturonases from *Aspergillus niger*. Biotechnol. Appl. Biochem..

[33] Munir M, Abdullah R, Haq IU, Kaleem A, Iqtedar M, Ashraf S (2020). Purification, characterization, kinetics and thermodynamic analysis of polygalacturonase from *Aspergillus tamarii* for industrial applications. Rev. Mex. Ing. Quím..

[34] Massa C, Degrassi G, Devescovi G, Venturi V, Lamba D (2007). Isolation, heterologous expression and characterization of an endopolygalacturonase produced by the phytopathogen *Burkholderia cepacia*. Protein Exp. Purif..

[35] Tounsi H, Sassi AH, Romdhane ZB, Lajnef M, Dupuy J-W, Lapaillerie D (2016). Catalytic properties of a highly thermoactive polygalacturonase from the mesophilic fungus *Penicillium occitanis* and use in juice clarification. J. Mol. Catal. B-Enzym..

[36] Pan X, Li K, Ma R, Shi PJ, Huang HQ, Yang PL (2015). Biochemical characterization of three distinct polygalacturonases from *Neosartorya fischeri* P1. Food Chem..

[37] Gundogdu M, Ozrenk K, Ercisli S, Kan T, Kodad O, Hegedus A (2014). Organic acids, sugars, vitamin C content and some pomological characteristics of eleven hawthorn species (*Crataegus* spp.) from Turkey. Biol. Res..

[38] Byaruagaba-Bazirake GW, Ransburg PV, Kyamuhangire W (2012). Characteristics of enzyme-treated banana juice from three cultivars of tropical and subtropical Africa. Afr. J. Food Sci. Technol..

[39] Ali ZM, Chin LH, Lazan H (2004). A comparative study on wall degrading enzymes, pectin modifications and softening during ripening of selected tropical fruits. Plant Sci..

[40] Xu H, Zhang PF, Zhang YC, Liu ZB, Zhang XB, Li ZM (2020). Overexpression and biochemical characterization of an Endo-α-1,4-polygalacturonase from *Aspergillus nidulans* in *Pichia pastoris*. Int. J. Mol. Sci..

[41] Zhu SY, Xu Y, Yu XW (2020). Improved homologous expression of the acidic lipase from *Aspergillus niger*. J. Microbiol. Biotechnol..

[42] Kumar S, Sharma HK, Sarkar BC (2011). Effect of substrate and fermentation conditions on pectinase and cellulase production by *Aspergillus niger* NCIM 548 in submerged (SmF) and solid state fermentation (SSF). Food Sci. Biotechnol..

[43] Jeong EB, Kim SA, Shin KC, Oh DK (2020). Biotransformation of protopanaxadiol-type ginsenosides in Korean ginseng extract into food-available compound K by an extracellular enzyme from *Aspergillus niger*. J. Microbiol Biotechnol..

[44] Kondaveeti S, Pagolu R, Patel SKS, Kumar A, Bisht A, Das D (2019). Bioelectrochemical detoxification of phenolic compounds during enzymatic pre-treatment of rice straw. J. Microbiol. Biotechnol..

[45] Barman S, Sit N, Badwaik LS, Deka SC (2015). Pectinase production by *Aspergillus niger* using banana (*Musa balbisiana*) peel as substrate and its effect on clarification of banana juice. J. Food Sci. Technol..

[46] Yang C, Wang JH, Chio CL, Chen XT, Zhang L, Zhang JN (2020). Low-cost recycling production of pectinase to increase the yield and quality of Muzao jujube juice by *Aspergillus niger*. Biofuel. Bioprod. Bioref..

[47] Pel HJ, de Winde JH, Archer DB, Dyer PS, Hofmann G, Schaap PJ (2007). Genome sequencing and analysis of the versatile cell factory *Aspergillus niger* CBS 513.88. Nat. Biotechnol..

