# Supplementary material for: A Novel Endo-Polygalacturonase from Penicillium oxalicum: Gene Cloning, Heterologous Expression and Its Use in Acidic Fruit Juice Extraction
Source: J Microbiol Biotechnol. 2022 Jan 7;32(4):464–72. doi: 10.4014/jmb.2112.12023 (PMC9628815; doi:10.4014/jmb.2112.12023)
Supplement: Supplementary file 1 [file jmb-32-4-464-supple.pdf]

https://doi.org/10.4014/jmb.2112.12023

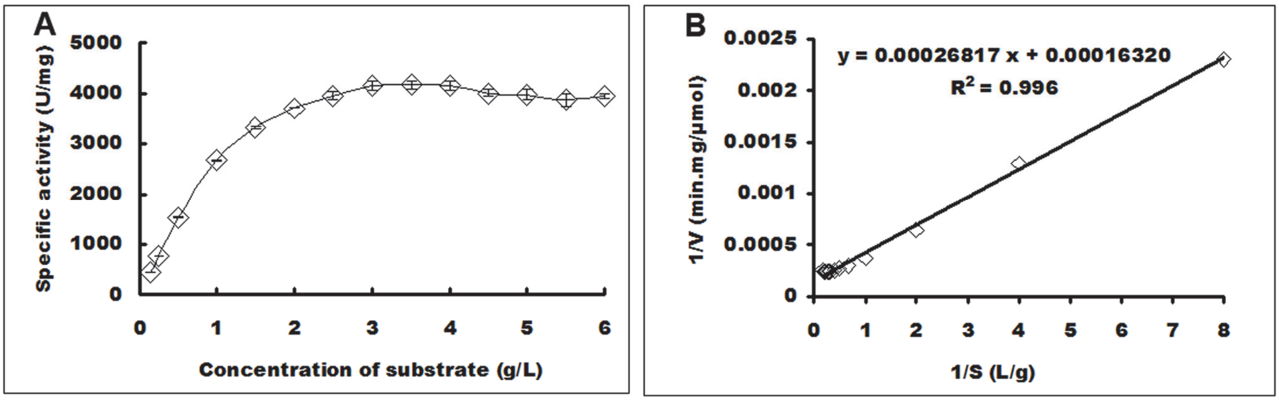

FIG S1. Michaelis-Menten Constant of PoxaEnPG28C. The kinetic parameters of the purified PoxaEnPG28C were determined by assaying the reaction rates for polygalacturonic acid at concentrations ranging from 0.125–6.0 g/L under the standard assay conditions (pH 4.5, 45°C and for 10 min) (A); error bars represent the standard deviation of three repeats. The Michaelis-Menten constant (K_m) and the maximum reaction velocity (V_{max}) were obtained from the Lineweaver-Burk plot (B).

TABLE S1. Effects of 2 mM metal ions on the enzyme activity of PoxaEnPG28C

Salts	Relative activity (%)[a]
Control	100.20±1.20
$BaCl_2$	80.88±1.76
$CaCl_2$	74.08±2.20
$CoCl_2$	95.00±1.47
$CuCl_2$	88.78±1.59
$FeCl_2$	92.27±3.10
KCl	89.54±5.53
$MgCl_2$	97.95±3.12
$MnCl_2$	39.12±1.36
NaCl	90.89±2.78
NiCl	79.53±2.57
$ZnCl_2$	87.91±1.23

[a]All experiments were performed in triplicate and the mean values are presented. The experiments were repeated three times and similar results were obtained.
